# Molecular phylogenetic analyses reveal multiple long-distance dispersal events and extensive cryptic speciation in *Nervilia* (Orchidaceae), an isolated basal Epidendroid genus

**DOI:** 10.3389/fpls.2024.1495487

**Published:** 2025-02-20

**Authors:** Stephan W. Gale, Jihong Li, Somran Suddee, Paweena Traiperm, Craig I. Peter, Tomas Buruwate, Benjamin J. Crain, Melissa K. McCormick, Dennis F. Whigham, Arni Musthofa, Khyanjeet Gogoi, Katsura Ito, Yukio Minamiya, Tatsuya Fukuda, Sven Landrein, Tomohisa Yukawa

**Affiliations:** ^1^ Flora Conservation Department, Kadoorie Farm and Botanic Garden, Hong Kong, Hong Kong SAR, China; ^2^ Department of National Parks, Forest Herbarium, Wildlife and Plant Conservation, Bangkok, Thailand; ^3^ Department of Plant Science, Faculty of Science, Mahidol University, Bangkok, Thailand; ^4^ Department of Botany, Rhodes University, Grahamstown, Makhanda, South Africa; ^5^ Mariri Environmental Centre L5 South Concession, Niassa Special Reserve, Mozambique; ^6^ North American Orchid Conservation Center, Smithsonian Environmental Research Center, Edgewater, MD, United States; ^7^ Integrated Research Laboratory, Faculty of Medicine, Public Health and Nursing, Universitas Gadjah Mada, Yogyakarta, Indonesia; ^8^ The Orchid Society of Eastern Himalaya, Daisa Bordoloi Nagar, Tinsukia, Assam, India; ^9^ Faculty of Agriculture and Marine Science, Kochi University, Monobeotsu, Nankoku, Kochi, Japan; ^10^ Tochigi Prefectural Museum, Utsunomiya, Japan; ^11^ Graduate School of Integrative Science and Engineering, Tokyo City University, Tokyo, Japan; ^12^ Tsukuba Botanical Garden, National Museum of Nature and Science, Tsukuba, Japan

**Keywords:** Asia monsoon, diversification, hysteranthy, lower Epidendroideae, out-of-Africa, species complex

## Abstract

**Introduction:**

The terrestrial orchid genus *Nervilia* is diagnosed by its hysteranthous pattern of emergence but is nested among leafless myco-heterotrophic lineages in the lower Epidendroideae. Comprising ca. 80 species distributed across Africa, Asia and Oceania, the genus remains poorly known and plagued by vague and overlapping species circumscriptions, especially within each of a series of taxonomically intractable species complexes. Prior small-scale, exploratory molecular phylogenetic analyses have revealed the existence of cryptic species, but little is otherwise understood of origin, the scale and timing of its biogeographic spread, or the palaeoclimatic factors that have shaped its ecology and given rise to contemporary patterns of occurrence.

**Methods:**

Here, we sample widely throughout the generic range, including 45 named taxa and multiple accessions referable to several widespread ‘macrospecies’, as well as material of equivocal identity and probable undescribed status, for the first time enabling an evaluation of taxonomic boundaries at both species and sectional level. Using nuclear (*ITS*) and plastid (*matK*, *trnL-F*) sequence data, we conduct phylogenetic (maximum parsimony and Bayesian inference) and ancestral area analysis to infer relationships and resolve probable origin and colonisation routes.

**Results:**

The genus is strongly supported as monophyletic, as are each of its three sections. However, the number of flowers in the inflorescence and other floral characters are poor indicators of sectional affinity. Dated ancestral area analysis supports an origin in Africa in the Early Oligocene, with spread eastwards to Asia occurring in the Late Miocene, plausibly via the Gomphotherium land bridge at a time when it supported woodland and savanna ecosystems.

**Discussion:**

Taxonomic radiation in Asia within the last 8 million years ties in with dramatic Himalayan-Tibetan Plateau uplift and associated intensification of the Asia monsoon. Multiple long-range migrations appear to have occurred thereafter, as the genus colonised Malesia and Oceania from the Pliocene onwards. The bulk of contemporary species diversity is relatively recent, potentially explaining the ubiquity of cryptic speciation, which leaves numerous species overlooked and unnamed. Widespread disjunct species pairs hint at high mobility across continents, extinction and a history of climate-induced vicariance. Persistent taxonomic challenges are highlighted.

## 1 Introduction

The Old World terrestrial orchid genus *Nervilia* Comm. ex Gaudich. is the sole member of subtribe Nerviliinae and the largest genus of tribe Nervilieae (Pridgeon et al., 2005; Chase et al., 2015; POWO, 2024), which is thought to have diverged from its sister tribe Gastrodieae ca. 35 million years ago (Mya; Li et al., 2019). Both tribes are nested within the ‘lower Epidendroid’ clade of the Epidendroideae, which with >21,160 accepted species is the largest orchid subfamily, accounting for more than three-quarters of all orchid diversity (Freudenstein and Chase, 2015). A consensus phylogenetic framework for the lower Epidendroids remains wanting, owing in part to the prevalence of myco-heterotrophy in the basal-most lineages, leading to high substitution rates in, and gene loss from, their plastid genomes (Rothacker, 2007; Górniak et al., 2010; Feng et al., 2016). This has complicated sequence alignment, confounded phylogenetic resolution and undermined stable classification (Chase et al., 2003; Lam et al., 2018). To an extent, whole plastome sequencing has helped clarify basal Epidendroid relationships (Li et al., 2019; Wen et al., 2022), but incongruence between nuclear and plastid trees remains a persistent challenge to the interpretation of evolutionary data sets and the attainment of a reliable taxonomy (Pérez-Escobar et al., 2021). As the only autotrophic member of its tribe and one of relatively few autotrophic lineages at the base of the subfamily, clearer understanding of patterns in speciation and trends in biogeographic occurrence in *Nervilia* could help shed light on the evolution of the lower Epidendroids as a whole (Chase et al., 2003, 2015; Pérez-Escobar et al., 2021).

*Nervilia* is diagnosed by its hysteranthous mode of emergence, by which separate generative (flower-bearing) and vegetative (leaf-bearing) shoots sprout in succession, typically with little or no overlap between the two (Pettersson, 1991; Gale et al., 2018). All emergent parts die back at the end of the growing season, with only the subterranean corm perennating through the winter or dry season to the next (Gale et al., 2021). This annual cycle and correspondingly ephemeral above-ground phase – an adaptation thought to have arisen in response either to marked seasonality in rainfall (Pettersson, 1991) or to an interplay of factors including temperature and resource limitation (Howard and Cellinese, 2020) – renders plants easily overlooked in the field and has led to the erroneous claim that some species are leafless myco-heterotrophs (Pettersson, 1991). Combined with their diminutive habit, sporadic occurrence and rarity in many cases, these attributes mean that most species remain poorly known. In fact, because flowers and leaves are rarely present at the same time, herbarium specimens tend to comprise just one or the other, and as two or more species may occur at the same site (Pettersson, 1991; Gale et al., 2015), shoots belonging to different species, or even to different genera, are sometimes mismatched on the same sheet (Pettersson, 1990; Gale et al., 2016, 2021; Ketjarun et al., 2019).

The genus occurs in tropical and subtropical Africa and Madagascar, Asia, Australasia and parts of Micronesia, Melanesia and Polynesia (Pridgeon et al., 2005), and is presently thought to contain in the region of 80 species (POWO, 2024). Tanzania, Thailand and Indonesia appear to be the countries with the greatest diversity, each with 12 or more species, but the majority of species are Asian (Gale et al., 2022; POWO, 2024). Given this geographic bias, Pettersson (1991) hypothesised that the genus originated in Asia. Even so, there are a number of reasons why biogeographic understanding of species diversity might be considered incomplete. Firstly, the very limited herbarium material available means that species circumscriptions and boundaries remain poorly resolved. Many pre-20th century names were published with superficial protologues that did not document morphological details now known to be important for species delimitation (Gale et al., 2007, 2015), but type material of these small, generally membranous plants is delicate and often badly preserved (Pettersson, 1990). This issue is especially problematic for the species of section *Linervia*, which possess just one flower, restricting options for the observation and analysis of floral traits (Gale et al., 2007). As a result, many names have been misapplied or later proven to be synonyms of incompletely known taxa (Seidenfaden and Smitinand, 1959–1965; Gale et al., 2010; Nusbauer et al., 2011; Gale and Watthana, 2014). Secondly, the lack of any range-wide or continental-scale revision of the genus, other than for the African species (Pettersson, 1991), means that, in the absence of a standard reference, taxonomic confusion has been propagated through the piecemeal misuse and repeated misinterpretation of names in regional or national treatments (Chen and Gale, 2009; Gale et al., 2007). In fact, the only attempt to critically compare all members of the genus known at the time dates back to the early 20th century, when the global tally stood at 37 species plus seven insufficiently known taxa (Schlechter, 1911).

**Figure 1:**
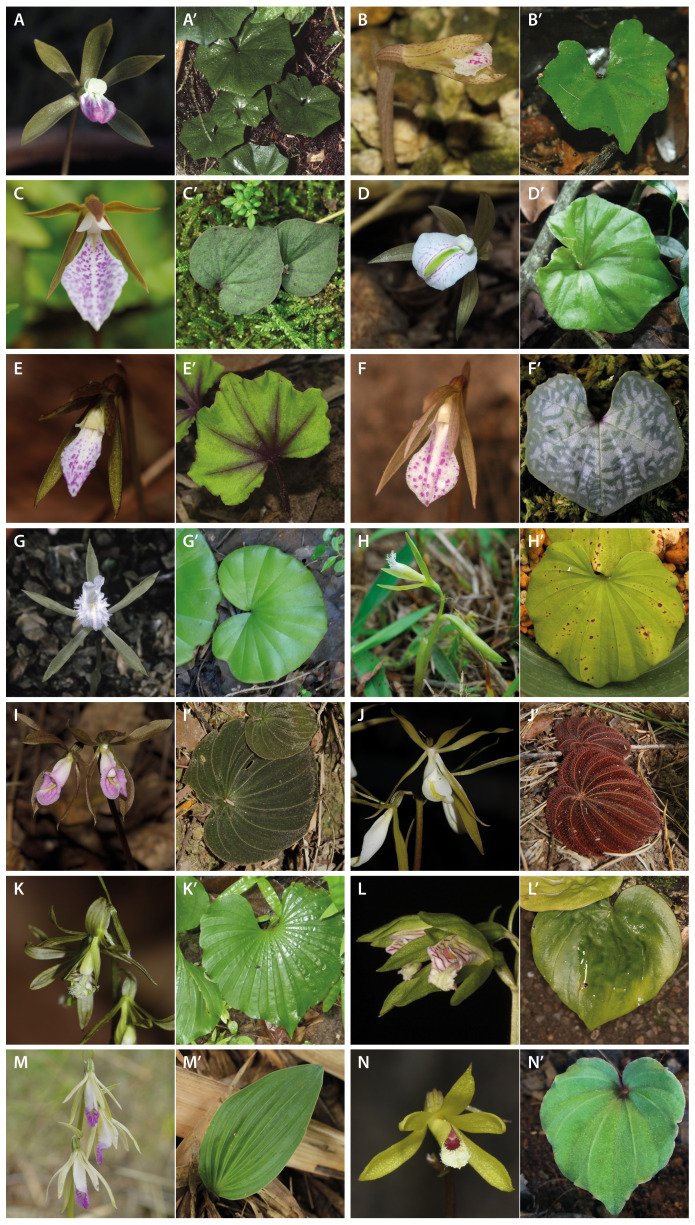
Morphological conservatism and sectional division in *Nervilia*. **(A–H’)** Section *Linervia*. **(I–J’**) Section *Vinerlia*. **(K–N’)** Section *Nervilia*. **(A, A’)** Flower and leaf of *N. adolphi* var. *adolphi* in Tanzania. **(B, B’)** Flower and leaf of *N. alisanensis* in China (Hainan). **(C, C’)** Flower and leaf of *N. juliana* in India. **(D, D’)** Flower and leaf of *N. khaoyaica* in Thailand. **(E, E’)** Flower of *N. mackinnonii* in Thailand and leaf of *N.* cf. *mackinnonii* (sample MY73 in **Table 1**) in Myanmar. **(F, F’)** Flower and leaf of *N. taiwaniana* in Taiwan. **(G, G’)** Flower and leaf of *N. simplex* in Malawi. **(H, H’)** Flowers and leaf of *N. cumberlegei* in Taiwan. **(I, I’)** Flowers and leaf of *N. plicata* in China (Hong Kong). **(J, J’)** Flowers and leaf of *N. plicata* in Thailand. **(K, K’)** Flowers and leaf of *N. concolor* in Thailand. **(L, L’)** Flowers and leaf of *N. kotschyi* var. *kotschyi* in Kenya. **(M, M’)** Flowers and leaf of *N. campestris* (=*N. holochila*) in Indonesia. **(N, N’)** Flower and leaf of *N. maculata* in Thailand.

To complicate matters further, *Nervilia* has been shown to contain a series of species complexes, each characterised by vegetative uniformity and only subtle differences in floral morphology that can nevertheless conceal wide genetic, cytological and biogeographic divergence and thus cryptic diversity (Gale et al., 2010, 2015, 2016, 2018; Ketjarun et al., 2019). Species complexes have been identified in all three presently accepted sections on the genus, but the so-called ‘*N. adolphi–punctata* alliance’ of section *Linervia* is the largest, with 30 or more species distributed throughout the generic range (Gale et al., 2015, 2018). Indeed, 21 of the 22 names published in *Nervilia* as new species since 2010 are referable to this taxonomically challenging complex on account of their one flower with a narrow, predominantly white and usually crimson-spotted, three-lobed lip and glabrous, cordate-polygonal leaf (**Figures 1A–F´**). This surge in species discovery reinforces that comprehensive taxonomic understanding remains some way off, especially in Asia, where all these new taxa were found. Gale et al. (2016) and Ketjarun et al. (2019) have highlighted that cryptic taxa may also occur in sections *Nervilia* and *Vinerlia*, notably within the widespread and polymorphic ‘macrospecies’ *N. concolor* (Blume) Schltr. and *N. plicata* (Andrews) Schltr., respectively.

All species produce just one inflorescence and one leaf per annual growth cycle, with the number of flowers borne by the inflorescence, as well as the size, outline and indumentum of the leaf, supposedly varying discretely among the three sections (Schlechter, 1911; Pettersson, 1991; Gale et al., 2021; **Figure 1**). Thus, as traditionally circumscribed, section *Nervilia* comprises plants with a four- or more-flowered scape and a comparatively large, glabrous, orbicular leaf; section *Vinerlia* comprises plants with a two-flowered scape and a pubescent, ovate-reniform leaf; and section *Linervia* comprises plants with a one-flowered scape and a small, cordate-polygonal or reniform leaf that is usually glabrous but which, in some species, is setose (Schlechter, 1911; Pettersson, 1991). However, there are a number of species that do not conform to this sub-division. For example, Pettersson (1991) reasoned that, despite its normally one-flowered scape and ovate leaf, *N. ballii* G.Will. is best placed in section *Nervilia* on account of its lip with a recurved mid-lobe and nectar guides, as is *N. shirensis* (Rolfe) Schltr., which is two- or three-flowered. On the other hand, Seidenfaden and Smitinand (1959–1965) noted that, despite its two- or three-flowered inflorescence, *N. cumberlegei* Seidenf. & Smitinand has a fimbriate lip much like the one-flowered *N. prainiana* (King & Pantl.) Seidenf. and *N. crispata* (Blume) Schltr. ex K.Schum. & Lauterb., both of which are now generally included in the synonymy of *N. simplex* (Thouars) Schltr. of section *Linervia* (**Figures 1G–H´**). And, acknowledging wide infraspecific variation, Pettersson (1990, 1991) assigned *N. kotschyi* (Rchb.f.) Schltr. to section *Nervilia* on account of details of the lip and tepals, even though its usually two-flowered scape and cordate-reniform leaf with fringed keels might justify its placement in section *Vinerlia* (**Figures 1L, L´**). Gale et al. (2021) postulated that details of floral anatomy, rather than flower number, will ultimately prove incisive in defining sectional identity.

To date, only two small-scale attempts have been made to integrate molecular phylogenetic data into analyses of species relationships in the genus (Gale et al., 2015, 2018). Employing nuclear (*ITS*) and plastid (*matK* and *trnL-F*) sequences, those studies uncovered surprisingly wide vegetative variation within some narrowly distributed species on the one hand, as well as broad uniformity in overall morphology among genetically and biogeographically distinct taxa on the other. But few species were sampled and most were from seasonal tropical Asia. So, although that work flagged cryptic speciation as a feature of the genus and hinted at divergence that reflects the morphology-based sectional classification, numerous ambiguities remain, particularly with regards to interpreting species taxonomy in the light of occurrence and ecology (Gale et al., 2010, 2015, 2018, 2021; Niissalo et al., 2020). For the first time, the present study samples throughout the generic range to enable the validity of the three sections to be tested. In doing so, we assess origin, examine how palaeoecological history has driven diversification and biogeographic spread, and explore the relationship between genetic divergence and morphological differentiation within each of the species complexes.

## 2 Materials and methods

### 2.1 Taxon sampling

Owing to the difficulty in positively identifying *Nervilia* species in the absence of correctly matched flowers and leaves, we included a mix of both named and unnamed accessions to account for as wide a cross section of the genus as possible and so permit an examination of patterns of genetic disparity among morphologically similar plants. Further, we included two or more accessions from different locations for some species in order to test vague or questionable species boundaries, particularly the taxonomically problematic ‘macrospecies’ *N. infundibulifolia* Blatt. & McCann, *N. simplex*, *N. plicata* and *N. concolor*, which have been subject to unstable and sometimes conflicting interpretation in different parts of their widespread geographic ranges. To help assess the merit of morphology-based assumptions within variable taxa, three names presently treated as synonyms by POWO (2024) were maintained for the purposes of this study: *N. campestris* (J.J.Sm.) Schltr. [now placed under *N. holochila* (F.Muell.) Schltr.], *N. carinata* (Roxb.) Schltr. (now placed under *N. concolor*) and *N. prainiana* (now placed under *N. simplex*).

In total, 96 *Nervilia* plants were sampled (**Table 1**). These included 86 samples representing 45 named taxa, four samples that could only be doubtfully referred to a particular named species and were thus qualified with “conferatur” [*viz N.* cf. *mackinnonii* (Duthie) Schltr., *N.* cf. *viridis* S.W.Gale, Watthana & Suddee and *N.* cf. *concolor*], and a further six samples that could not be matched with any published species and were thus suspected to represent undescribed taxa. Our sampling covered all three sections of the genus, including several taxa with a flower number ‘atypical’ of the section in which they are placed (**Table 1**). Also included was *N. stolziana* Schltr. which, on account of its spurred lip, was previously assigned (together with *N. pectinata* P.J.Cribb, not included in this study) to section *Kyimbilaea*, which has since been subsumed under section *Linervia* (Pettersson, 1991). In all, we included 11 taxa (13 samples) from Africa and Madagascar, 30 species (60 samples) from the seasonal Asian tropics, seven species (nine samples) from the moist Asian tropics, and seven species (14 samples) from Oceania. Based on the phylograms presented by Chase et al. (2015) and Freudenstein and Chase (2015), we included one sample each of *Corymborkis veratrifolia* (Reinw.) Blume (Tribe Tropidieae), *Monophyllorchis maculata* Garay (Tribe Triphoreae) and *Gastrodia peichatieniana* S.S.Ying (Tribe Gastrodieae) as outgroups from subfamily Epidendroideae for phylogenetic analysis, plus one sample of *Habenaria dentata* (Sw.) Schltr. (Tribe Orchideae) as an additional outgroup from subfamily Orchidoideae for biogeographic analysis. All samples were collected and transported with permission [CITES permits: ROP-008-2018, ROP-045-2019, PCIP-20-00094, CA-307/2012, 2014-TH006062/CA, 2021-TH010447/BE, JPHTN/PPP/BO-100-24/1(40), 003/16-01, 008/16-01].

**Table 1:** Collection localities, jurisdiction, voucher specimens and GenBank accession numbers for the samples used in this study.

Taxon (flower number)	Jurisdiction	Region	Sample code	Voucher specimen (herbarium)	GenBank accession number		
					*ITS*	*matK*	*trnL-F*
Section *Linervia*							
*Nervilia adolphi* var. *adolphi* (1)	Tanzania, Mbeya Region	Africa & Madagascar	–	*B. Pettersson et al. 449* (K)	PQ512847	PQ514079	PQ510141
*Nervilia alishanensis* (1)	Taiwan, Chiayi County	Seasonal tropical Asia	TAR1	*C.-I. Chen s.n.* (MBK)	KM892985	KM986829	KM892999
*Nervilia alishanensis* (1)	China, Hainan Province	Seasonal tropical Asia	GAL2009028	*S. Gale 2009028* (IBSC)	KM892987	KM986837	KM892997
*Nervilia borneensis* (1)	Malaysia, Sabah State	Moist tropical Asia	–	*A. Lamb AL2089/2011* (SAN)	PQ512849	PQ514082	PQ510145
*Nervilia cumberlegei* (2–3)	Taiwan, Chiayi County	Seasonal tropical Asia	–	*C.-I. Chen & M.-S. Sai C* (MBK)	KM892994	KM986835	KM893007
*Nervilia futago* (1)	Japan, Miyazaki Prefecture	Seasonal tropical Asia	HAS1	*T. Yukawa 05-66* (TNS)	HQ848247	HQ848209	HQ848167
*Nervilia futago* (1)	Japan, Okinawa Prefecture	Seasonal tropical Asia	YAN1	*S. Gale et al. 14* (MBK)	HQ848243	HQ848205	HQ848163
*Nervilia hemratii* (1)	Thailand, Kanchanaburi Province	Seasonal tropical Asia	–	*N. Tetsana et al. 2222* (BKF)	PQ512860	PQ514096	PQ510158
*Nervilia infundibulifolia* (1)	China, Yunnan Province	Seasonal tropical Asia	SG1316	*Q. Liu 153718* (HITBC)	MG452037	MG452070	MG452105
*Nervilia infundibulifolia* (1)	Thailand, Kanchanaburi Province	Seasonal tropical Asia	NER03	*S. Duangjai 03052015* (BKF)	MG452035	MG452068	MG452103
*Nervilia infundibulifolia* (1)	Thailand, Kanchanaburi Province	Seasonal tropical Asia	NER30	*C. Ngernsaengsaruay s.n.* (BKF)	MG452036	MG452069	MG452104
*Nervilia infundibulifolia* (1)	Laos, Xayabouri Province	Seasonal tropical Asia	HNL-KFBG 0776	*S. Gale & P. Sysouphanthong HNL-KFBG 0776* (HNL)	PQ512861	PQ514097	PQ510159
*Nervilia infundibulifolia* (1)	Vietnam, Dak Lak Province	Seasonal tropical Asia	AL262	*L. Averyanov & T. Maisak AL262* (HN)	PQ512862	PQ514098	PQ510160
*Nervilia juliana* (1)	India, Assam State	Seasonal tropical Asia	–	*K. Gogoi 0047* (GUBH)	–	PQ514099	PQ510161
*Nervilia kasiensis* (1)	Laos, Vientiane Province	Seasonal tropical Asia	–	*S. Gale et al. HNL-KFBG 0537* (HNL)	PQ512863	PQ514100	PQ510162
*Nervilia khaoyaica* (1)	Thailand, Nakhon Ratchasima Province	Seasonal tropical Asia	SG021	*P. Triphetch 120368* (BKF)	–	MG452071	MG452106
*Nervilia khaoyaica* (1)	Thailand, Nakhon Ratchasima Province	Seasonal tropical Asia	SG012	*S. Gale s.n.* (QBG)	PQ512864	PQ514101	PQ510163
*Nervilia lanyuensis* (1)	Taiwan, Taitung County	Seasonal tropical Asia	–	*S.-W. Chung s.n.* (TAIF)	KM892983	KM986834	KM892998
*Nervilia lilacea* (1)	Tanzania, Iringa Region	Africa & Madagascar	Y1373	*B. Pettersson et al. 153* (K)	PQ512867	PQ514104	PQ510167
*Nervilia lilacea* (1)	Malawi, Southern Region	Africa & Madagascar	SG017	*B. Pettersson & A. Gassner 359* (K)	–	–	PQ510166
*Nervilia mackinnonii* (1)	Nepal, Bagmati Province	Seasonal tropical Asia	SG007	*B. Raskoti 196* (TUCH)	–	KM986836	KM893008
*Nervilia mackinnonii* (1)	Thailand, Tak Province	Seasonal tropical Asia	NSC05-01	*S. Chanhormhual 05* (BKF)	MG452050	MG452084	MG452119
*Nervilia* cf. *mackinnonii*	Myanmar, Mandalay Division	Seasonal tropical Asia	MY73	*N. Tanaka et al. 036189* (MBK)	PQ512854	PQ514086	PQ510150
*Nervilia macroglossa* (1)	Nepal, Bagmati Province	Seasonal tropical Asia	–	*B. Raskoti 271* (KATH)	KM892984	KM986833	KM893005
*Nervilia marmorata* (1)	Thailand, Chiang Rai Province	Seasonal tropical Asia	NER14	*S. Duangjai 250314* (BKF)	MG452040	MG452074	MG452109
*Nervilia marmorata* (1)	Thailand, Chiang Rai Province	Seasonal tropical Asia	NER29	*S. Suddee 4910* (BKF)	MG452045	MG452079	MG452114
*Nervilia muratana* (1)	Vietnam, Quang Binh Province	Seasonal tropical Asia	–	*L. Averyanov et al. HAL12510* (HN)	MG452048	MG452082	MG452117
*Nervilia nipponica* (1)	Japan, Kochi Prefecture	Seasonal tropical Asia	KL12-1	*S. Gale FOK067838* (MBK)	HQ848232	HQ848195	HQ848155
*Nervilia nipponica* (1)	South Korea, Jeju Island	Seasonal tropical Asia	SG1	*N.S. Lee D459* (EWH)	HQ848251	HQ848213	HQ848174
*Nervilia palawensis* (1)	Palau, Babeldaob Island	Oceania	84L	*B. Crain 143* (US)	PQ512870	PQ514107	PQ510170
*Nervilia palawensis* (1)	Palau, Ngerekebesang Island	Oceania	112L	*B. Crain 214* (US)	PQ512869	PQ514106	PQ510169
*Nervilia petraea* (1)	Mozambique, Niassa Province	Africa & Madagascar	–	*T. Buruwate s.n.* [OrchidMAP 10069*]	–	PQ514108	PQ510171
*Nervilia prainiana* (1)	Laos	Seasonal tropical Asia	–	*T. Yukawa 1205* (TNS)	PQ512880	PQ514119	PQ510181
*Nervilia punctata* (1)	Indonesia, East Java Province	Moist tropical Asia	–	*J. Comber 1114* (K)	MG452065	–	MG452136
*Nervilia simplex* (1)	Nepal, Bagmati Province	Seasonal tropical Asia	SG005	*B. Raskoti 270* (KATH)	–	PQ514121	PQ510183
*Nervilia simplex* (1)	Thailand, Chiang Rai Province	Seasonal tropical Asia	NER04	*S. Duangjai 120614* (BKF)	MG452033	MG452066	MG452101
*Nervilia simplex* (1)	Madagascar, Ihorombe Region	Africa & Madagascar	K-DNA Bank 31433	*D. Roberts 554* (K)	PQ512881	PQ514122	PQ510184
*Nervilia simplex* (1)	China, Yunnan Province	Seasonal tropical Asia	SG1318	*Q. Liu 153717* (HITBC)	PQ512882	PQ514123	PQ510185
*Nervilia simplex* (1)	Laos, Xayaboury Province	Seasonal tropical Asia	HNL-KFBG 0754	*S. Gale et al. HNL-KFBG 0754* (HNL)	PQ512883	PQ514124	PQ510186
*Nervilia singaporensis* (1)	Singapore, Bukit Timah	Moist tropical Asia	–	*M.A. Niissalo SING2019-1365* (SING)	MT152902	–	MT152903
*Nervilia stolziana* (1)	Malawi, Northern Region	Africa & Madagascar	–	*B. Pettersson et al. 48* (K)	–	–	PQ510191
*Nervilia tahanshanensis* (1)	Taiwan, Pingtung County	Seasonal tropical Asia	–	*H.-C. Hung 001* (KBCC)	PQ512889	PQ514130	PQ510192
*Nervilia taiwaniana* (1)	Taiwan, Taitung County	Seasonal tropical Asia	CSA1	*C.-I. Chen & M.-S. Sai A* (MBK)	KM892989	KM986842	KM893002
*Nervilia taiwaniana* (1)	Taiwan, Takao County	Seasonal tropical Asia	G5A	*S. Gale, 2007015* (MBK)	KM892990	KM986838	KM893003
*Nervilia trangensis* (1)	Thailand, Trang Province	Seasonal tropical Asia	NER02	*S. Suddee 4647* (BKF)	MG452060	MG452093	MG452129
*Nervilia trangensis* (1)	Thailand, Trang Province	Seasonal tropical Asia	NTK01	*S. Duangjai 300317* (BKF)	MG452061	MG452095	MG452131
*Nervilia trichophylla* (1)	Palau, Aulupse’el Island	Oceania	–	*F.R. Fosberg 47551* (US)	–	–	PQ510193
*Nervilia umphangensis* (1)	Thailand, Tak Province	Seasonal tropical Asia	SG1210	*P. Prommanut 308* (BKF)	MG452062	MG452096	MG452132
*Nervilia umphangensis* (1)	Thailand, Tak Province	Seasonal tropical Asia	SS4731	*S. Suddee 4731* (BKF)	MG452063	MG452097	MG452133
*Nervilia viridis* (1)	Thailand, Chiang Mai Province	Seasonal tropical Asia	SG1331	*S. Watthana & P. Momkaew 4199* (BKF)	MG452064	MG452100	MG452135
*Nervilia viridis* (1)	China, Yunnan Province	Seasonal tropical Asia	SG1317	*Q. Liu & S. Gale 153716* (HITBC)	PQ512890	PQ514131	PQ510194
*Nervilia* cf. *viridis*	Thailand, Nakhon Ratchasima Province	Seasonal tropical Asia	NER27	*S. Duangjai 020815* (BKF)	MG452054	MG452098	MG452123
*Nervilia* cf. *viridis*	Thailand, Nakhon Ratchasima Province	Seasonal tropical Asia	NER01	*S. Duangjai 020815* (BKF)	MG452052	MG452086	MG452121
*Nervilia* sp. *nov. 1*	Japan, Okinawa Prefecture	Seasonal tropical Asia	–	*M. Yokota s.n.* (RYU)	PQ512884	PQ514125	PQ510187
*Nervilia* sp. *nov. 2*	Solomon Islands, Malaita Province	Oceania	–	*S. Gale et al. SIMB38* (MBK)	PQ512885	PQ514126	PQ510188
*Nervilia* sp. *nov. 3*	Solomon Islands, Malaita Province	Oceania	–	*S. Gale et al. SIMB49* (MBK)	PQ512886	PQ514127	PQ510189
*Nervilia* sp. *nov. 4*	Indonesia, Bali Province	Moist tropical Asia	–	*T. Yukawa 1746* (TNS)	PQ512887	PQ514128	–
*Nervilia* sp. *nov. 5*	Nepal	Seasonal tropical Asia	–	*T. Yukawa 1022* (TNS)	KM892982	KM986832	KM893001
Section *Vinerlia*							
*Nervilia platychila* (2–3)	Federated States of Micronesia, Yap Island	Oceania	00505651	*M. Falanruw & M. Faimau 5670* (US)	–	PQ514109	–
*Nervilia platychila* (2–3)	Palau, Babeldaob Island	Oceania	151L	*B.J. Crain* 209 (US)	PQ512872	PQ514111	PQ510173
*Nervilia platychila* (2–3)	Palau, Babeldaob Island	Oceania	173L	*B.J. Crain* 209 (US)	PQ512873	PQ514112	PQ510174
*Nervilia platychila* (2–3)	Palau, Ngerekebesang Island	Oceania	220L	*B.J. Crain* 209 (US)	PQ512871	PQ514110	PQ510172
*Nervilia plicata* (2, rarely 3)	Indonesia, Sulawesi Island	Moist tropical Asia	InNp1	*P. Leong s.n.* (SING)	PQ512874	PQ514113	PQ510175
*Nervilia plicata* (2, rarely 3)	Taiwan, Chiayi County	Seasonal tropical Asia	CSB1	*C.-I. Chen & M.-S. Sai B* (MBK)	KM892995	KM986841	KM893006
*Nervilia plicata* (2, rarely 3)	China, Yunnan Province	Seasonal tropical Asia	Y0919	*T. Yukawa 0919* (TNS)	PQ512875	PQ514114	PQ510176
*Nervilia plicata* (2, rarely 3)	Malaysia, Sabah State	Moist tropical Asia	SG011	*A. Lamb AL2090/2011* (SAN)	PQ512876	PQ514115	PQ510177
*Nervilia plicata* (2, rarely 3)	Nepal, Lumbini Province	Seasonal tropical Asia	SG010	*B. Raskoti 204* (TUCH)	PQ512877	PQ514116	PQ510178
*Nervilia plicata* (2, rarely 3)	Thailand, Kanchanaburi Province	Seasonal tropical Asia	NER16	*S. Duangjai 120416* (BKF)	MG452049	MG452083	MG452118
*Nervilia plicata* (2, rarely 3)	Myanmar	Seasonal tropical Asia	Y1204	*T. Yukawa 1204* (TNS)	PQ512878	PQ514117	PQ510179
*Nervilia plicata* (2, rarely 3)	China, Hong Kong	Seasonal tropical Asia	SG1143	*S. Gale 1143* (KFBG)	PQ512879	PQ514118	PQ510180
Section *Nervilia*							
*Nervilia ballii* (1, rarely 2)	Malawi, Southern Region	Africa & Madagascar	Nball	*B. Pettersson 299* (K)	–	PQ514080	PQ510142
*Nervilia ballii* (1, rarely 2)	Mozambique, Niassa Province	Africa & Madagascar	SG1663	*T. Buruwate s.n.* [OrchidMAP 10071**]	–	PQ514081	PQ510143
*Nervilia bicarinata* (2–12)	Madagascar, Ihorombe Region	Africa & Madagascar	–	*D. Roberts 542* (K)	PQ512848	–	PQ510144
*Nervilia campestris* (2–4)	Indonesia, Yogyakarta Province	Moist tropical Asia	–	*A. Musthofa 01* (BO)	PQ512850	–	PQ510146
*Nervilia carinata* (1–5)	Nepal, Lumbini Province	Seasonal tropical Asia	SG003	*B. Raskoti300* (KATH)	PQ512851	PQ514083	PQ510147
*Nervilia carinata* (1–5)	Myanmar, Mandalay Division	Seasonal tropical Asia	MyNa73	*W.H. Khin 024044* (MBK)	PQ512852	PQ514084	PQ510148
*Nervilia concolor* (4–18)	Palau, Peleliu Island	Oceania	00505610	*A. Rinehart LR22619* (US)	–	PQ514092	–
*Nervilia concolor* (4–18)	Japan, Okinawa Prefecture	Seasonal tropical Asia	OkNa1023	*S. Gale et al. 1* (MBK)	PQ512855	PQ514090	PQ510153
*Nervilia concolor* (4–18)	Malaysia, Sabah State	Moist tropical Asia	SG001	*A. Lamb AL2091/2011* (SAN)	PQ512856	PQ514091	PQ510154
*Nervilia concolor* (4–18)	India, Karnataka State	Seasonal tropical Asia	00320003	*C. Saldanha & T.P. Ramamoorthy 474* (US)	–	–	PQ510151
*Nervilia concolor* (4–18)	Tonga, Tongatapu Island	Oceania	00320010	*Wilkes s.n.* (US)	–	–	PQ510152
*Nervilia concolor* (4–18)	Guam, Tamuning Village	Oceania	00505621	*L. Raulerson 14145* (US)	–	PQ514087	–
*Nervilia concolor* (4–18)	Philippines, Rizal Province	Moist tropical Asia	68020102	*M. Ramos 22683* (US)	–	PQ514088	–
*Nervilia concolor* (4–18)	Society Islands, Tetiaroa Atoll	Oceania	00505750	*F.R. Fosberg 54579* (US)	–	PQ514089	–
*Nervilia* cf. *concolor*	Taiwan	Seasonal tropical Asia	Y0978	*T. Yukawa 0978* (TNS)	PQ512853	PQ514085	PQ510149
*Nervilia fordii* (4–10)	Laos, Xayaboury Province	Seasonal tropical Asia	HNL-KFBG 0599	*S. Gale et al. HNL-KFBG 0599* (HNL)	PQ512857	PQ514093	PQ510155
*Nervilia fordii* (4–10)	Laos, Xekong Province	Seasonal tropical Asia	HNL-KFBG 1119	*S. Gale et al. HNL-KFBG 1119* (HNL)	PQ512858	PQ514094	PQ510156
*Nervilia fordii* (4–10)	Thailand, Nakhon Ratchasima Province	Seasonal tropical Asia	TS02	*T. Sando 02* (BKF)	MG452047	MG452081	MG452116
*Nervilia gammieana* (5–8)	India, Uttarakhand State	Seasonal tropical Asia	–	*S. Deva 7557* (C)	PQ512859	PQ514095	PQ510157
*Nervilia holochila* (3–4)	Australia	Oceania	–	[GenBank]	AF324178	–	–
*Nervilia kotschyi* var. *kotschyi* (2–8)	Kenya, Mombasa County	Africa & Madagascar	2013-G-255	*B. Schlumpberger 2013-G-255* (Herrenhäuser Gärten)	PQ512865	PQ514102	PQ510164
*Nervilia kotschyi* var. *purpurata* (2–8)	South Africa, Mpumalanga Province	Africa & Madagascar	SG1659	*D. McMurtry 15172* (HSMC)	PQ512866	PQ514103	PQ510165
*Nervilia maculata* (1–2)	Thailand, Tak Province	Seasonal tropical Asia	–	*S. Suddee et al. 5157* (BKF)	PQ512868	PQ514105	PQ510168
*Nervilia renschiana* (3–8)	Mozambique, Niassa Province	Africa & Madagascar	–	*T. Buruwate s.n.* [OrchidMAP 10068***]	–	PQ514120	PQ510182
*Nervilia shirensis* (2–3)	Nigeria, Oyo State	Africa & Madagascar	–	*M.W. Chase 9057* (K)	AF521066	AY121735	AF519945
*Nervilia* sp. *nov. 6*	Thailand	Seasonal tropical Asia	–	*T. Yukawa 1208* (TNS)	PQ512888	PQ514129	PQ510190
Outgroups							
*Corymborkis veratrifolia*	Malaysia, Sabah State	–	–	*A. Kocyan AK981020-1-01* (Z)	PQ512843	PQ514075	–
*Gastrodia peichatieniana*	China, Hong Kong	–	HK43268	*S. Gale s.n.* (KFBG)	PQ512844	PQ514076	–
*Habenaria dentata*	China, Hong Kong	–	KFBG2126A	*S. Gale SG1009* (KFBG)	PQ512845	PQ514077	PQ510139
*Monophyllorchis maculata*	South America	–	–	*T. Yukawa 0764* (TNS)	PQ512846	PQ514078	PQ510140

Sectional affiliation is indicated, as is the number of flowers present on the scape of each species. “-” under GenBank accession number indicates sequence unavailable.

*https://vmus.adu.org.za/vm_view_record.php?vm=OrchidMAP-10069.

**https://vmus.adu.org.za/vm_view_record.php?vm=OrchidMAP-10071.

***https://vmus.adu.org.za/vm_view_record.php?vm=OrchidMAP-10068.

### 2.2 DNA extraction, PCR and sequencing

Of the 100 samples used in this study, 63 were newly sequenced, either from fresh material (48 samples) or from well-preserved herbarium specimens (15 samples). Total DNA was extracted using a QIAGEN DNeasy^®^ plant DNA kit (Hilden, Germany) according to the manufacturer’s instructions. The internal transcribed spacer (*ITS*) region of nuclear ribosomal DNA was amplified using the primers of White et al. (1990) or Sun et al. (1994), the plastid maturase K gene (*matK*) region (including part of the flanking *trnK* introns) was amplified in three sections using the primers of Hidayat et al. (2005), and the entire *trnL-F* region (comprising the *trnL* intron and the *trnL-F* intergenic spacer) was amplified using the c and f primers of Taberlet et al. (1991). PCR was performed in a total reaction mixture of 25 µl containing 1 µl of template DNA (2–10 ng), 5 µl of 5 × Phire^®^ reaction buffer with MgCl_2_, 0.5 µl 10 mM of dNTP mix, 0.5 µl of Phire^®^ hot start II DNA polymerase (Finnzymes, Finland) and 10 pmol of each primer (Beijing Genomics Institute). The thermal cycler programme consisted of an initial denaturation step at 98°C for 30 s, followed by 35 cycles of 5 s at 98°C, 5 s at 60°C for *ITS*, 10 s at 55°C for *matK* and 5 s at 55°C for the *trnL-F* region, 20 s at 72°C, and a final extension at 72°C for 1 min. Amplification products were purified using a DNA purification Kit (Beijing Genomics Institute). Purified PCR products were sequenced using an ABI 3730 DNA Sequencer (Applied Biosystems, Foster City, California). All sequences have been deposited in GenBank (**Table 1**).

### 2.3 Phylogenetic analysis

Alignments were constructed using the MAFFT multiple alignment plugin in Geneious v11.1.4 (Kearse et al., 2012), with subsequent adjustment by eye. We excluded two poly-A regions comprising 41 and 61 positions in the *trnL–F* and *matK* genes, respectively (**Supplementary File S1**). An incongruence length difference (ILD) test (Farris et al., 1995) was performed in PAUP* v4.0b10 (Swofford, 2003) to assess whether the individual *matK* and *trnL–F* data sets, and the *ITS* and combined cpDNA data sets (**Supplementary File S1**), reflect similar potential phylogenies; 1,000 replicates, each with 1,000 random addition sequence replicates and tree bisection-reconnection (TBR) branch swapping, were performed in each test, and a *P* value of <0.05 was considered significant (Sullivan, 1996; Darlu and Lecointre, 2002). A “hard” incongruence test was also performed by directly comparing respective topologies, as well as resolution, for each clade generated in the separate analyses, with bootstrap percentages (BP) of ≥85% (Chase et al., 2000) and posterior probabilities (PP) of ≥0.95 (Martínez-Azorín et al., 2011) being taken as evidence of strong support.

Both the homogeneity test for the *matK* and *trnL-F* data sets (*P* = 0.881) and visual node-by-node comparisons of trees generated for either region individually revealed no major topological disparities for nodes of BP ≥85% and PP ≥0.95, and so the two ptDNA regions were combined. Tree topologies generated for the individual *ITS* and ptDNA data sets using Bayesian inference (BI) were also largely congruent with those using maximum parsimony (MP; **Supplementary File S2**). However, the ILD test indicated significant incongruence between the *ITS* and ptDNA data sets (*P* = 0.001). Even so, visual comparison of the trees generated from the two data sets uncovered no topological disparities with nodes of BP ≥85% and PP ≥0.95, except for the position of a single clade containing four samples representing three species [*N. bicarinata* (Blume) Schltr., *N. kotschyi* and *N. shirensis*; **Supplementary File S2**]. Since Cunningham (1997) and Yoder et al. (2001) have argued that combined data sets improve phylogenetic accuracy regardless of incongruence, and numerous phylogenetic studies have found that trees generated from combined data sets with or without samples responsible for topological disparities remain highly consistent (e.g. Li et al., 2011; Kumar et al., 2022), we concatenated the *ITS* and ptDNA data sets and interpreted the resulting combined phylograms.

Phylogenetic analysis of individual and multilocus alignments were carried out using MP in PAUP* v4.0b10 and BI in MrBayes v3.2 (Huelsenbeck and Ronquist, 2003). For MP analyses, heuristic searches were conducted with 1,000 random addition replicates followed by TBR branch swapping. All characters were unordered and equally weighted with gaps (including unavailable sequences) treated as missing data. Topological robustness was assessed using 1,000 bootstrap replicates. For BI analyses, each DNA region was assigned its own model of nucleotide substitution, as determined by the Akaike information criterion (AIC) in Modeltest v3.06 (Posada and Crandall, 1998). Four simultaneous Monte Carlo Markov Chains (MCMC) were run, with sampling one tree every 1,000 generations for 30,000,000 generations, starting with a randomly generated tree. Majority rule (>50%) consensus trees were constructed after removing the first 25% of sampled trees as burn-in.

### 2.4 Ancestral area reconstruction

In constructing a dated phylogenetic tree, a single accession was selectively retained for each taxon represented by more than one sample (**Supplementary File S3**). Divergence times were estimated using a Bayesian uncorrelated relaxed-clock model implemented in BEAST 2.7.6 (Bouckaert et al., 2019) with priors placed on the node for tribes Nervilieae and Gastrodieae (offset 34.93 Mya, mean:1, sigma:1) and the node for subfamilies Epidendroideae and Orchidoideae (offset 64 Mya, mean:1, sigma:1), based on results presented by Givnish et al. (2015); Li et al. (2019) and Li et al. (2022). MCMC searches were run for 50,000,000 generations and sampled every 5,000 generations, with convergence being monitored using Tracer 2.7.6 (Bouckaert et al., 2019). The effective sample sizes (ESSs) of all parameters were assessed as more than 200 and the maximum clade credibility tree was computed using treeAnnotator 2.7.6 (Bouckaert et al., 2019).

Four areas of endemism were defined for biogeographic analysis, reflecting the extant distribution of *Nervilia* demarcated by Pridgeon et al. (2005) as well as the climatic zones discernible within this range based primarily on seasonality, which is presumed to be of importance for the hysteranthous habit (Gale et al., 2021): (area 1) tropical Africa & Madagascar, (area 2) seasonal (monsoonal) tropical Asia, (area 3) aseasonal, moist tropical Asia, and (area 4) Oceania (encompassing Australasia, Micronesia, Melanesia and Polynesia). Ancestral area reconstruction was then performed using the package BioGeoBEARS (Matzke, 2016) in R 4.3.2 (R Core Team, 2023), applying the dispersal–extinction–cladogenesis (DEC) model (Ree and Smith, 2008), ML version of Dispersal Vicariance Analysis (DIVALIKE; Ronquist, 1997) and Bayesian biogeographical inference model (BAYAREALIKE; Landis et al., 2013) with the maximum range-size parameter set to three. We tested each of these models with and without founder-event speciation, which was incorporated with J-parameter modelling jump dispersal (Matzke, 2016). All six permutations were compared using likelihood values, and Akaike information criterion (AIC) was performed in BioGeoBEARS using the maximum clade credibility tree from the BEAST analyses described above. The best-fit model was selected based on lower corrected Akaike information criterion (AICc) values with larger weight (wAICc), representing relative support for each model (Burnham and Anderson, 2002). All underlying raw data used in the phylogenetic analyses and ancestral area reconstruction are available in the Dryad Digital Repository, DOI: 10.5061/dryad.tb2rbp0bn.

## 3 Results

Sequence data for all three regions (*ITS*, *matK* and *trnL-F*) were newly generated for 41 samples, chloroplast data (*matK* and *trnL-F*) were generated for a further seven samples, *ITS* and *matK* data were generated for three samples, *ITS* and *trnL-F* data were generated for two samples, and *matK* or *trnL-F* data were individually generated for a further five samples (**Table 1**). Statistics relating to the aligned matrix for each region and for the combined data sets are shown in **Table 2**.

**Table 2:** Statistics relating to the phylogenetic data sets used in this study.

Information	*ITS*	*matK*	*trnL–F*	Combined ptDNA data sets (*matK* & *trnL–F*)	Combined *ITS* and ptDNA data sets
No. ingroups	78	86	89	95	96
No. outgroups	3	3	1	3	3
Aligned length	735	1,797	1,116	2,913	3,648
No. variable characters	139	232	94	326	465
No. parsimony-informative characters	304	278	190	468	772
Tree length	1008	761	395	1,162	2,204
Consistency index (CI)	0.685	0.756	0.82	0.774	0.721
Retention index (RI)	0.932	0.952	0.962	0.954	0.941
Rescaled consistency index (RC)	0.638	0.719	0.789	0.738	0.679
Best-fit model determined by AIC in Modeltest	GTR+I+G	GTR+G	GTR+G	GTR+G	GTR+G

### 3.1 Phylogenetic analysis

**Figure 2:**
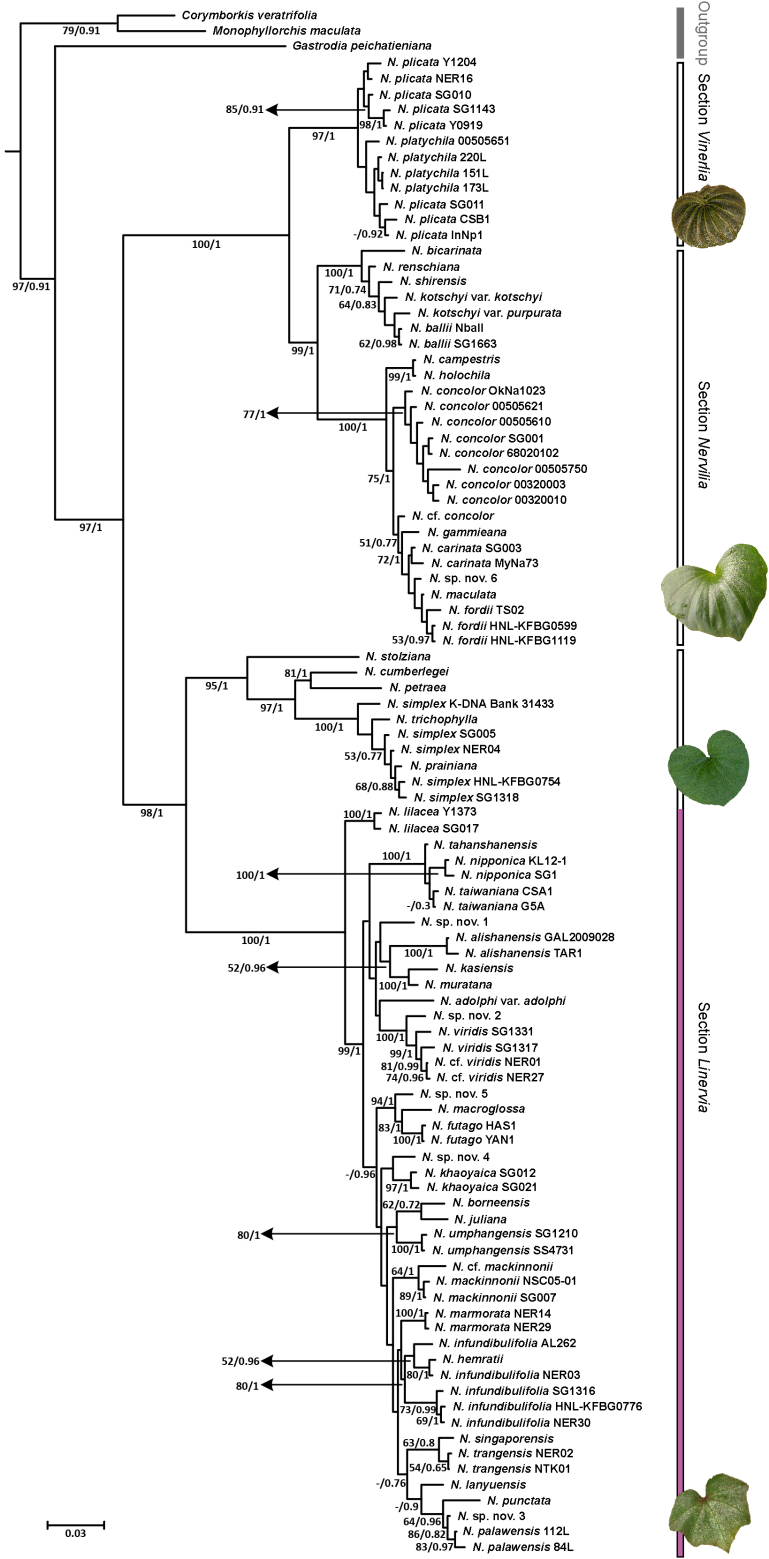
Phylogram obtained from Bayesian inference (BI) analysis of the combined *ITS* and ptDNA data sets. Numbers at the nodes indicate bootstrap percentages and Bayesian posterior probabilities, respectively. “-” indicates that the node collapsed in MP analysis. Sectional affiliation is indicated at right, as is the extent of the species-rich *Nervilia adolphi*-*punctata* alliance (shown in purple).

The genus *Nervilia* in its entirety was strongly supported as monophyletic, whether assessed using combined (BP 97%, PP 1.00; **Figure 2**) or individual *ITS* and ptDNA data sets (**Supplementary File S2**). In the combined tree (**Figure 2**), all ingroup taxa fell into two strongly supported clades, one comprising section *Linervia* (BP 98%, PP 1.00) and the other composed of sections *Vinerlia* and *Nervilia* together (BP 100%, PP 1.00). Represented here by *N. plicata* and *N. platychila* Schltr., section *Vinerlia* was also supported as monophyletic (BP 97%, PP 1.00), as was section *Nervilia* (BP 99%, PP 1.00). Section *Nervilia* itself comprised two well resolved sub-clades, one containing five African species [*N. bicarinata*, *N. renschiana* (Rchb.f) Schltr., *N. shirensis*, *N. kotschyi* and *N. ballii*; BP 100%, PP 1.00] and the other containing seven named species [*N. campestris*, *N. holochila*, *N. concolor*, *N. gammieana* (Hook.f.) Pfitzer, *N. carinata*, *N. maculata* (C.S.P.Parish & Rchb.f.) Schtlr. and *N. fordii* (Hance) Schtlr.] plus *N.* cf. *concolor* and *N.* sp. nov. 6 from Asia and Oceania (BP 100%, PP 1.00). Within the latter, *N. campestris* and *N. holochila* together formed a single, strongly supported lineage (BP 99%, PP 1.00) that was sister to all remaining members of this sub-clade (BP 75%, PP 1.00), which in turn partitioned the eight positively identified *N. concolor* accessions (BP 77%, PP 1.00) as separate from an unresolved grade of nine samples representing *N. gammieana*, *N. carinata*, *N. maculata*, *N. fordii*, *N.* cf. *concolor* and *N.* sp. nov. 6.

Section *Linervia* was also composed of two strongly supported sub-clades, one comprising *N. stolziana*, *N. cumberlegei*, *N. petraea* (Afzel. ex Sw.) Summerh. and the various samples belonging to the *N. simplex* complex, including *N. prainiana* and *N. trichophylla* Fukuy. (BP 95%, PP 1.00), and the other containing 24 other named species plus two unverified accessions (*N.* cf. *viridis* and *N.* cf. *mackinnonii*) and five putatively undescribed species (BP 100%, PP 1.00), all of which are referable to the ‘*N. adolphi–punctata* alliance’ on the basis of leaf and floral characters. Within this latter sub-clade, the two *N. lilacea* Jum. & H.Perrier accessions from Africa (BP 100%, PP 1.00) were strongly supported as sister to all other accessions from Asia and Oceania (BP 99%, PP 1.00). A derived internal sub-clade of the latter containing *N. tahanshanensis* T.P.Lin & W.M.Lin, *N. nipponica* Makino and *N. taiwaniana* S.S.Ying also received strong support (BP 100%, PP 1.00), as did a sister relationship between *N. kasiensis* S.W.Gale & Phaxays. and *N. muratana* S.W.Gale & S.K.Wu (BP 100%, PP 1.00). Similarly, the undescribed *N.* sp. nov. 2 from the Solomon Islands was strongly supported as sister to a clade containing the two Thai and Chinese *N. viridis* samples plus the two Thai *N.* cf. *viridis* accessions (BP 100%, PP 1.00), with the monophyly of the latter four also being strongly supported (BP 99%, PP 1.00). Further, the pair of samples included for each of *N. nipponica*, *N. alishanensis* T.C.Hsu, S.W.Chung & C.M.Kuo, *N. futago* S.W.Gale & T.Yukawa, *N. khaoyaica* Suddee, Watthana & S.W.Gale, *N. umphangensis* Suddee, Rueangr. & S.W.Gale, *N. mackinnonii*, *N. marmorata* S.W.Gale, Suddee & Duangjai and *N. palawensis* Schtlr. all formed strongly supported clades; but this was not the case for the pair of *N. trangensis* S.W.Gale, Suddee & Duangjai and *N. taiwaniana* samples. Meanwhile, the five *N. infundibulifolia* samples formed a clade inclusive of *N. hemratii* S.W.Gale, Tetsana & Suddee (BP 80%, PP 1.00), among which posterior probabilities hinted at a degree of internal structure.

### 3.2 Biogeographic analysis

**Figure 3:**
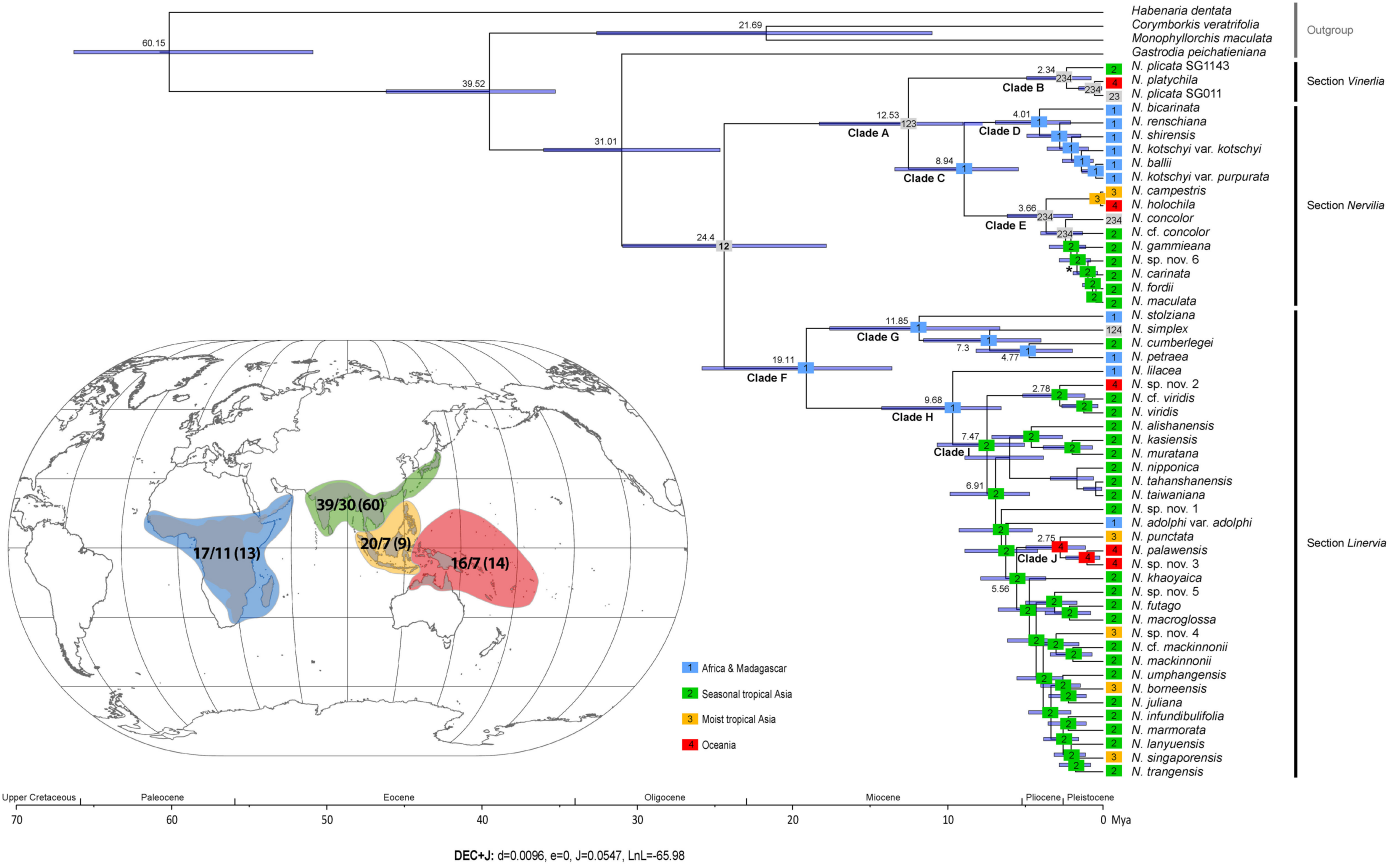
Spatio-temporal reconstruction of *Nervilia* according to the best-fit model (DEC+J) inferred by BioGeoBEARS and the maximum clade credibility tree obtained by BEAST analysis. The blue bar at each node indicates the 95% credibility intervals with mean node ages (Mya) shown above. Coloured rectangles correspond to the four biogeographic regions defined for the purposes of this study: blue (1) indicates Africa & Madagascar, green (2) seasonal tropical Asia, orange (3) moist tropical Asia and red (4) Oceania. Grey boxes indicate taxa that span two or more biogeographic regions. The worldwide occurrence of *Nervilia* is shown inset (grey shaded area), with the coloured polygons corresponding to the four biogeographic regions. Numbers shown in each polygon indicate the total number of *Nervilia* species known in that region according to POWO (2024) on the left of the forward-slash, followed by the number of species sampled in this study and the total number of samples in brackets.

Estimated divergence times for the genus encompassing 52 taxa are presented in **Figure 3**. The results support a close relationship between the tribes Nervilieae (represented by *Nervilia*) and Gastrodieae (represented by *Gastrodia* R.Br.), with divergence between the two genera placed at around 31.01 Mya in the Early Oligocene, with a 95% highest posterior density (HPD) interval ranging from 18.5 to 38.45 Mya. The common ancestral age of *Nervilia* was inferred to be 24.4 Mya (95% HPD 14.77–34.93 Mya). Ancestral lineage Clade A diverged at 12.53 Mya (95% HPD 6.24–25.14 Mya), giving rise to two derived lineages, one representing section *Vinerlia* (Clade B) and the other representing section *Nervilia* (Clade C). Clade C subsequently split at 8.94 Mya (95% HPD 3.98–20.88 Mya), generating clades D and E, each of which underwent further differentiation at 4.01 Mya (95% HPD 1.52–11.08 Mya) and 3.66 Mya (95% HPD 1.16–11.31 Mya), respectively. Ancestral lineage Clade F, representing section *Linervia*, split at 19.11 Mya (95% HPD 10.31–30.76 Mya) into two derived clades, G and H, each of which underwent further divergence at 11.85 Mya (95% HPD 3.81–24.07 Mya) and 9.68 MYA (95% HPD 5.47–21.63 Mya), respectively. Diversification of all derived lineages within the genus occurred from the Late Miocene onwards but this process was concentrated in the Pliocene and appears to have persisted into the Pleistocene (**Figure 3**).

BioGeoBEARS analysis identified the DEC (dispersal–extinction–cladogenesis) +J model as the best-fit model, recovering the lowest AICc value (138.5) and highest wAICc value (0.69) of the six models considered (**Table 3**). This model suggests that the ancestral *Nervilia* population had a distribution encompassing either Africa & Madagascar (area 1) or seasonal tropical Asia (area 2), and that it diverged into two distinct lineages, Clades A and F, in the Late Oligocene (**Figure 3**). Species derived from the earliest settlers of Clade A eventually gave rise to the common ancestor of Clades B and C in the Middle Miocene. Clade C appears to have originated in Africa & Madagascar, with subsequent divergence in the Late Miocene leading to diversification in that region during the Pliocene (Clade D), as well as contemporaneous dispersal to seasonal and moist tropical Asia (areas 2 and 3) initially, followed by Oceania (area 4) in the Pliocene (Clade E). Most diversification in the latter appears to have occurred in seasonal tropical Asia, but the rapid colonisation of seasonal and moist tropical Asia as well as Oceania by *N. concolor* over this timeframe is noteworthy. In contrast, diversification of Clade B occurred entirely outside of Africa & Madagascar, with its spread firstly across seasonal tropical Asia being followed by more recent colonisation of moist tropical Asia and Oceania during the Pleistocene.

**Table 3:** BioGeoBEARS estimation of ancestral areas for *Nervilia*.

Model	LnL	Number of parameters	Parameters				AICc model weight
			*d*	*e*	*j*	AICc	
DEC	-79.55	2	0.026	0.0097	0	163.3	2.70E-06
**DEC+J**	**-65.98**	**3**	**0.0096**	**1.00E-12**	**0.055**	**138.5**	**0.69**
DIVALIKE	-73.47	2	0.028	2.00E-09	0	151.2	0.0012
DIVALIKE+J	-66.79	3	0.015	1.00E-12	0.044	140.1	0.31
BAYAREALIKE	-103.8	2	0.031	0.089	0	211.8	8.20E-17
BAYAREALIKE+J	-72.05	3	0.0069	1.00E-07	0.068	150.6	0.0016

The row in bold indicates the best-fit model identified by BioGeoBEARS analysis.

The progenitor of Clade F was unequivocally of African & Madagascan origin, giving rise to two Clades, G and H, during the Early Miocene. Clade G underwent further differentiation primarily in the same region in the Middle to Late Miocene, but with the *N. simplex* lineage exhibiting enormous dispersal to seasonal tropical Asia and Oceania in the Late Miocene, mirroring the rapid spread of *N. concolor*. Clade H is also evidently of African & Madagascan origin and has the Afro-Malagasy *N. lilacea* at its base, but is otherwise characterised by dispersal to, and subsequent explosive diversification within, seasonal tropical Asia from the Late Miocene onwards, with several more recent, independent onward dispersals to both moist tropical Asia and Oceania. Clade J presents an interesting case of wide dispersal to and differentiation across the Pacific Islands, with subsequent colonisation of moist tropical Asia, from the late Pliocene onwards. *Nervilia adolphi*, in contrast, appears to represent an unusual dispersal back to Africa in the Late Miocene.

Overall, several long-distance dispersal events are revealed in the evolution of the genus. These include at least three independent migrations from Africa & Madagascar to seasonal tropical Asia in Clades C, G and H, and multiple colonisations of moist tropical Asia from seasonal tropical Asia in Clade I and possibly Clade E. Dispersal to Australasia and the Pacific appears to represent the most recent biogeographic step, having occurred both from seasonal tropical Asia in Clade I (and possibly Clade B) and from moist tropical Asia in Clade E. Moist tropical Asia was also colonised from Oceania in Clade J and, as already noted, Africa was ‘re-colonised’ from seasonal tropical Asia in Clade I.

## 4 Discussion

As has previously been surmised (Dressler, 1993; Pettersson, 1991; Chase et al., 2003, 2015; Górniak et al., 2010; Freudenstein and Chase, 2015), *Nervilia* is here resolved as monophyletic and phylogenetically isolated, with our results adding to mounting evidence of a close affiliation with *Gastrodia* at tribal level (Li et al., 2019; Pérez-Escobar et al., 2021). Although broader relationships among the basal-most Epidendroids remain contentious, the diminutive stature and ephemeral, often leafless habit of many of the constituent taxa renders them difficult to sample and challenging to analyse as compared with the generally showier and more robust higher Epidendroids (Li et al., 2018). The insights that our results provide are thus an important step in unraveling biogeographic trends and patterns in speciation across the grade. Though not yet exhaustive, the phylogenetic framework presented here sheds light on probable geographic origin and modes of dispersal and divergence, with ramifications for the evolution, biogeography, taxonomy and classification of *Nervilia*, a key lower Epidendroid genus.

### 4.1 First phylogenetic insights into the origin of *Nervilia*

*Nervilia* has been represented by up to just three samples in prior phylogenetic analyses of tribal- or genus-level relationships in the Orchidaceae (Rothacker, 2007; Li et al., 2019; Pérez-Escobar et al., 2021). Our findings corroborate a nested placement among the basal Epidendroids but, by virtue of much broader taxon sampling, we elaborate farther reaching hypotheses relating to the temporal and spatial scale of its evolution. Firstly, our analyses imply an origin in the Early Oligocene in either Africa & Madagascar or the seasonal Asian tropics, contrasting the one previous assertion by Pettersson (1991) that the genus arose in Asia, and most likely the wet Asian tropics. In fact, our results suggest that the genus was not present in that region until much more recently. Whilst further outgroup optimisation could yet alter this perspective, its affiliation with *Gastrodia* in the Gastrodieae on the one hand, and with *Epipogium* Borkh. and *Stereosandra* Blume in tribe Nervilieae itself on the other (Chase et al., 2015), might be expected to recover the same equivocal position, since all three genera are similarly widespread across the Old World tropics (Pridgeon et al., 2005). However, because Africa and Asia have never been geographically connected, it is necessary to discern which is the more likely ancestral area. In this regard, the prevalence of African branches at the more basal stem nodes (i.e. Clades C, F, G and H in **Figure 3**) lends weight to an African origin, all the more so for the generally eastward trajectory witnessed in the evolution of the genus as a whole: by and large, from Africa to seasonal tropical Asia, and from there onto the wet Asian tropics and Oceania. Movement from Africa to seasonal tropical Asia is also apparent in the transition from Clade H to Clade I in the Late Miocene (ca. 10 Mya onwards; **Figure 3**), a pattern probably repeated from Clade C to Clade E more or less contemporaneously and, given the absence of section *Vinerlia* in Africa (Pettersson, 1991), from Clade A to B over the same period, too.

The inferred timing of the origin of the genus is especially illuminating, since the Early Oligocene (from around 33.5 Mya onwards) was marked by the onset of an icehouse climate (Coxall and Pearson, 2007; Liu et al., 2009). This global transition is associated with significant sea-level drop, major aridification and a shift to more pronounced seasonality in rainfall as compared with the preceding warmer and more humid later Eocene (Berggren and Prothero, 1992; Miller et al., 2005; Guo et al., 2008). Although a more northerly inter-tropical convergence zone is thought to have delivered generally higher precipitation and thus wetter conditions to a band stretching across northern Africa and the Tethys oceans (Couvreur et al., 2021), the resulting expansion of savanna-like grassland in Africa and southern Eurasia, as well as subtropical woody savanna in central and southern China, is postulated to have led to fragmentation of closed tropical forest across these land masses (Morley, 2007; Guo et al., 2008). The advent of hysteranthy in the *Nervilia* lineage could thus be intrinsically linked to this period of increased seasonality in rainfall and lower mean temperatures, with the development of more open habitats at low to middle latitudes potentially offering distinct advantages for a terrestrial, seasonally dormant habit. Though the Early Oligocene is generally viewed as a time of widespread extinction of terrestrial biodiversity (Berggren and Prothero, 1992), compelling evidence for the first appearance of, and diversification within, numerous plant lineages at this time is accumulating (e.g. Zhou et al., 2012; Couvreur et al., 2021; Xue et al., 2024).

### 4.2 Both incremental inter-continental spread and long-range migration underpin the occurrence of *Nervilia* today

Progressive northward drift of the African plate through the Oligocene resulted in reconnection with Eurasia in the Middle Miocene (ca. 19–15 Mya) via formation of the Gomphotherium land bridge and eventual closure of the east Tethys Seaway ca. 14 Mya (Hamon et al., 2013; Couvreur et al., 2021). The Arabian plate, which had been contiguous with Africa throughout the Cenozoic and remained so at this juncture, is thought to have supported woodland and savanna ecosystems comprising warm and wet-adapted elements prior to undergoing aridification once in its modern position from the Late Miocene onwards (Steinthorsdottir et al., 2021). In light of this tectonic-cum-palaeoclimatic sequence and the phylogenetic chronology presented here, it seems reasonable to deduce that the very limited occurrence of *Nervilia* in the Arabian Peninsula today, with only the widespread Afro-Malagasy *N. bicarinata* being found in isolated parts of Yemen and Oman (Pettersson, 1991), is relictual and plausibly the result of climate-induced vicariance, as has been inferred in the biogeography of numerous sub-Saharan African lineages (Couvreur et al., 2021). The presence of *Nervilia* in seasonal tropical Asia within the last 10 Mya – apparently in the form of all three sections of the genus – might therefore be congruent with incremental spread through open woodland across the Gomphotherium land bridge to the Indian subcontinent, and from there to continental Southeast Asia, a pattern of incremental inter-continental migration that has been invoked in the dispersal of many ‘out-of-Africa’ palaeotropical taxa, including members of the disparate families Annonaceae, Asparagaceae and Hyacinthaceae (Zhou et al., 2012; Ali et al., 2013; Howard et al., 2022).

The prevailing occurrence of the African *Nervilia* species in deciduous and semi-deciduous forest, woodland savanna and grassland today (Pettersson, 1991) further hints towards an ancestral association with seasonally arid landscapes. In contrast, the species of topical Asia occur in a wider range of habitats, encompassing grassland and sparse forest types (Roxburgh, 1832; Su, 2000; Gale et al., 2014) but favouring closed-canopy communities, including semi-evergreen, mixed deciduous (or monsoon) and montane forest (e.g. Gale et al., 2015, 2018; Gale and Phaxaysombath, 2017), as well as true lowland rainforest (e.g. Smith, 1909, 1918). Expansion through continental and insular tropical Asia therefore appears to have gone hand-in-hand with extensive niche differentiation, including colonisation of the dark, moist, evergreen forest understorey. The recent discovery of partial mycoheterotrophy in *N. nipponica*, an Asian, forest-dwelling member of section *Linervia* in which reliance on fungal partners is most pronounced at lower light intensities (Nomura et al., 2013; Gale et al., 2021), invites closer scrutiny of the eco-physiological factors that could have facilitated this radiation, whether underpinned by vicariance or geodispersal. By combining carbon gain measurements with phylogenetic analysis of a cross-section of the genus representative of different habitat types in both Africa and Asia, it would be possible to address climate-linked landscape-scale patterns of divergence in light of the evolution of variable mixotrophy. But perhaps even more tellingly in this respect is the apparent loss of hysteranthy in a few derived Asiatic species – *N. borneensis* J.J.Sm., *N. muratana* and *N. kasiensis* – which produce successive, temporally overlapping flowering and leafing shoots along a persistent stolon (Smith, 1909; Gale and Wu, 2007; Gale and Phaxaysombath, 2017), implicating exceptional adaptive convergence that warrants finer phylogenetic reconstruction using next generation sequencing.

Whilst a comparatively ‘short hop’ overland from Africa to Arabia and onto seasonal tropical Asia via India therefore seems plausible and parsimonious in the palaeoclimatic contexts of the Middle to Late Miocene, our ancestral area analysis points to further, more complex patterns of migration thereafter. All three sections of the genus bear the same signature of recent arrival in moist tropical Asia and Oceania, as evidenced by the appearance within the past ca. 2.8 million years of section *Vinerlia* (represented here by *N. plicata* and *N. platychila*) in Malesia, Micronesia, New Caledonia and Fiji, section *Nervilia* (represented here by *N. concolor* and *N. campestris*/*N. holochila*) in Malesia, New Guinea, tropical Australia and the Southwest Pacific, and various species of section *Linervia* at various locations throughout this vast region. This timing broadly coincides with the Pliocene-Pleistocene boundary, a period of further global cooling, decreasing availability of growing season moisture and forest fragmentation (Couvreur et al., 2021; Steinthorsdottir et al., 2021). The geographic (and taxonomic) expansion of *Nervilia* across these land masses can probably be attributed at least in part to emergence of the Sunda shelf, since a terrestrial Sundaland was a consistent feature of the Cenozoic at least until the early Pliocene (5 Mya; Hall, 2009) with subsequent exposure occurring episodically through the Pleistocene (Voris, 2000; Sarr et al., 2019). However, permanent separation of Sundaland from both Wallacea and Oceania (Hall, 2009) implicates longer range onward dispersal in at least those lineages that gave rise to *N. platychila*, *N. holochila*, *N. palawensis* and, independently, two undescribed species (sp. nov. 2 and 3) both found on Malaita in the Solomon Islands, as well as in the lineage that gave rise to *N. punctata*, apparently through migration from Oceania ‘back’ to Malesia. Moreover, the surprising placement of the African *N. adolphi* within the overwhelmingly Asian ‘*N. adolphi–punctata* alliance’ of section *Linervia* is indicative of a somewhat deeper, long-range dispersal back to Africa, meriting further investigation of the origin and spread of the few other Afro-Malagasy members of this complex not included in this study (in particular, *N. fuerstenbergiana* Schltr. and *N. subintegra* Summerh.). Evidence of similar long-range dispersal from tropical Asia to Africa during the late Miocene has been uncovered in other plant groups with a marked Africa-Asia-Australasia disjunction (e.g. Li et al., 2009), though not yet, to our knowledge, in the Orchidaceae. We contend that the minute, mobile Orchidaceous dust seed could have been instrumental in facilitating both the stepwise spread and longer distance migrations uncovered here (McCormick and Jacquemyn, 2014; Givnish et al., 2016).

### 4.3 Recent diversification and the prevalence of cryptic species boundaries

One the most striking features in the evolution of the genus, however, is the enormous taxonomic diversification that appears to have occurred from around 8 Mya, notably in section *Linervia* and particularly in seasonal tropical Asia. This timing and regionalisation coincide with the most active phase in the uplift of the Himalaya-Tibetan Plateau, which precipitated significant intensification of the Indian and East Asian monsoons (Zhisheng et al., 2001). Replacing Oligocene subtropical aridity (Guo et al., 2008), the evolution of this atmospheric system is tightly correlated with phased Himalayan orogeny through the Miocene, transforming the geography and biology of the continent through alternating circulations of moist, oceanic air during the summer and dry, inland air during the winter (Li et al., 2015; Nguyen et al., 2024). This process is believed to have reached its zenith by around 3.6–2.6 Mya, although the East Asia winter monsoon continued to strengthen thereafter (Zhisheng et al., 2001). Given that our results reveal both ongoing speciation and independent but broadly synchronous dispersal events between subtropical and tropical Asia and Oceania in all three sections of the genus from the upper Pliocene well into the Pleistocene, it is probable that monsoonal oscillations over tropical East Asia and Oceania played an important role in this dynamism, providing further evidence of the role of seasonality in the evolution of the genus as a whole. A similar explanation was proposed by Ji et al. (2024) in interpreting patterns of diversification within certain lineages of the orchid tribe Collabieae, and especially in the genus *Calanthe* R.Br.

The apparent link between rapid, recent diversification and the ubiquity of cryptic taxa across the genus warrants deeper examination. All three sections contain species complexes (Gale et al., 2016, 2018; Ketjarun et al., 2019) but the present study reveals the enormous geographic scale of their spread, mostly from the Pliocene onwards. Cursory appraisal of the multi-flowered *N. concolor* and allies in tropical Asia and Oceania has led to considerable taxonomic discord, with the name *N. aragoana* having been widely applied across Asia and the Pacific (e.g., Lewis and Cribb, 1989; Pearce and Cribb, 2002; Gale and Watthana, 2014) before being subsumed under the synonymy of the former without detailed analysis (POWO, 2024). Whilst our results support the recognition of a single though variable species ranging from southern Japan to Borneo and from the Western Ghats to the Society Islands, the inclusion also of *N. carinata* in its synonymy is unfounded, demanding critical review of its circumscription with respect to certain names not sampled in our study [e.g. *N. scottii* (Rchb.f.) Schltr. and *N. tibetensis* Rolfe; POWO, 2024]. Indeed, it is clear that this alliance harbours unrecognised diversity, given the presence of both morphologically distinct (*N.* sp. nov. 6) and anomalous (*N.* cf. *concolor*) entities here. Moreover, the placement of the strikingly different – and one-flowered – *N. maculata* (**Figures 1N, N´**) in this clade underscores the need for caution before lumping grossly similar ‘floral types’ together without fully evaluating finer characters and phylogenetic distance in the light of ecological differentiation.

The same may apply to the macrospecies *N. plicata*, which is here found to comprise a grade of morphologically diverse, continental Southeast Asian and insular tropical Asian and Pacific elements, the latter including *N. platychila*, with a degree of structure suggested among some samples within each of these two vast regions hinting at a possible link between biogeographic history and taxonomic divergence (Ketjarun et al., 2019). But on an even more remarkable scale, species diversity within the *N. adolphi–punctata* alliance appears to have been generated across the entire generic range predominantly within the last ca. 8 million years, and much of it far more recently than that. Despite vegetative uniformity, this complex patently still conceals cryptic taxa, including the five undescribed species sampled here. In contrast, *N. simplex* exhibits little genetic discontinuity across its enormous range, supporting the incorporation of both the continental Southeast Asian *N. prainiana* and Micronesian *N. trichophylla* despite the morphological disparities that have been used to define them (e.g. Seidenfaden, 1978). Next generation sequencing, as well as analyses of polyploidy, reticulate evolution and possible hybridisation (Chennaveeraiah and Jorapur, 1966; Gale et al., 2015) and introgression, are recommended to further disentangle the evolutionary history of these taxonomically intractable lineages. In addition, since knowledge of pollination biology in *Nervilia* remains fragmentary (Pettersson, 1989; Gale, 2007), clarification of taxonomic, ecological and geographic biases in rewarding, deceptive and autogamous systems, for example, could further shed light on how floral divergence and pollinator shifts have shaped speciation and biogeographic spread (Ackerman et al., 2023).

### 4.4 Taxonomic implications

That said, sufficient clarity is already achieved to draw several taxonomic conclusions. Firstly, though flower number is confirmed as an unreliable basis for defining a sectional classification of the genus, the three presently recognised sections are nevertheless clearly natural. Section *Vinerlia* occurs only in Asia and Oceania, not Africa and Madagascar, and is typified by the widespread *N. plicata*. Higher resolution, integrated phylogenetic and morphological research is needed to ascertain whether *N. platychila* can be maintained as distinct from that species, possibly reflecting a biogeographic split between insular tropical Asia plus Oceania on the one hand, versus inland, continental Asia on the other. Though both are two-flowered, the pubescent, reniform leaf and longitudinally folded labellum are diagnostic. Examination of other species that probably belong here, including *N. ignobilis* Tuyama and *N. umenoi* Fukuyama, is needed to better define the section. Pridgeon et al. (2005) list *N. maculata* as the type of section *Vinerlia*, but that species is here unequivocally placed in section *Nervilia*.

Section *Nervilia* occurs throughout the range of the genus and is highly variable not only in flower number, but also in terms of leaf shape and indumentum, as well as floral morphology. Pettersson (1991) used two labellum characters to define the section in Africa – the presence of nectar guides and a recurved mid-lobe – but these do not apply outside that continent. He also referred to a possible distinction in pollination ecology, with Eumenid wasps known to pollinate two African species (*N. bicarinata* and *N. shirensis*), but no pollination studies have yet been conducted on Asian or Australasian members of the section to either confirm or refute this as a reliable sectional trait. Therefore, though the African species appear to be monophyletic and sister to all remaining members, section *Nervilia* lacks a clear synapomorphy at present. As concluded elsewhere (POWO, 2024), we confirm that *N. campestris* is most likely conspecific with *N. holochila*, presenting an intriguing case of vicariance across Wallace’s Line, albeit highly localised to Java on the western side. Despite wide morphological variation throughout its enormous range, *N. concolor* is monophyletic and there is little evidence of internal genetic structure. However, *N. carinata* is not conspecific.

Section *Linervia* comprises two natural sub-groups: the fimbriate-lipped species typified by the extremely widespread *N. simplex* plus the spurred African species (represented here by *N. stolziana*) previously placed in section *Kyimbilaea*, and those species with an entire labellum mid-lobe that constitute the *N. adolphi–punctata* alliance. Within the latter, all four *N. viridis* samples included here were found to be monophyletic and almost certainly conspecific, even though Gale et al. (2018) refrained from combining the two “*N.* cf. *viridis*” samples from eastern Thailand on the grounds that Bayesian coalescence analysis resolved them as distinct. Intriguingly, this continental Asiatic species falls sister to an unnamed species from the Solomon Islands. The Himalayan *N. macroglossa* and southern Japanese *N. futago* present another interesting disjunction, potentially alluding to historic extinction of other closely related, geographically contiguous taxa, as suggested by the selected BioGeoBEARS model. The widespread continental Asian *N. infundibulfolia* exhibits considerable internal genetic structure worthy of further examination but is monophyletic only if *N. hemratii* is considered synonymous. *Nervilia punctata* is here placed in a clade with the Micronesian *N. palawensis* and an unnamed species from the Solomon Islands. Our analyses corroborate Gale et al. (2018) in determining *N. punctata* to be Malesian, with prior records of this entity from continental Southeast Asia (e.g. Seidenfaden, 1978; Gale and Watthana, 2014) probably amounting to misidentifications of *N. mackinnonii* or other members of this problematic complex. Though the section is overwhelmingly one-flowered, the two- or rarely three-flowered *N. cumberlegei* also belongs here, and thus the only synapomorphy for the section appears to be the elongating fruiting scape (Pettersson, 1991; Gale et al., 2006).

## 5 Conclusions

The cumulative effects of multiple dispersal events coupled with isolation through extinction or vicariance here emerge as predominant drivers shaping the current geographical distribution of species within *Nervilia*. Africa is singled out as the probable ancestral centre (or ‘cradle’) of the genus as well as that of sections *Nervilia* and *Linervia*, whilst seasonal tropical Asia is identified as a radiative reservoir (or ‘museum’) of species diversity, especially for section *Linervia*, and probably gave rise to section *Vinerlia*. Despite the relatively ancient origin of the genus as a whole, speciation appears to have accelerated from the Late Miocene onwards, correlating to Himalayan uplift and intensification of the Asian monsoon. Other than the widespread macrospecies *N. concolor*, *N. plicata* and *N. simplex*, most species probably arose through speciation within areas, with high levels of regional endemism.

## Data Availability

The datasets presented in this study can be found in online repositories. The names of the repository/repositories and accession number(s) can be found in the article/**Supplementary Material**.
