# Supplementary figures and images for: Molecular phylogenetic analyses reveal multiple long-distance dispersal events and extensive cryptic speciation in Nervilia (Orchidaceae), an isolated basal Epidendroid genus
Source: Front Plant Sci. 2025 Feb 20;15:1495487. doi: 10.3389/fpls.2024.1495487 (PMC11883896; doi:10.3389/fpls.2024.1495487)

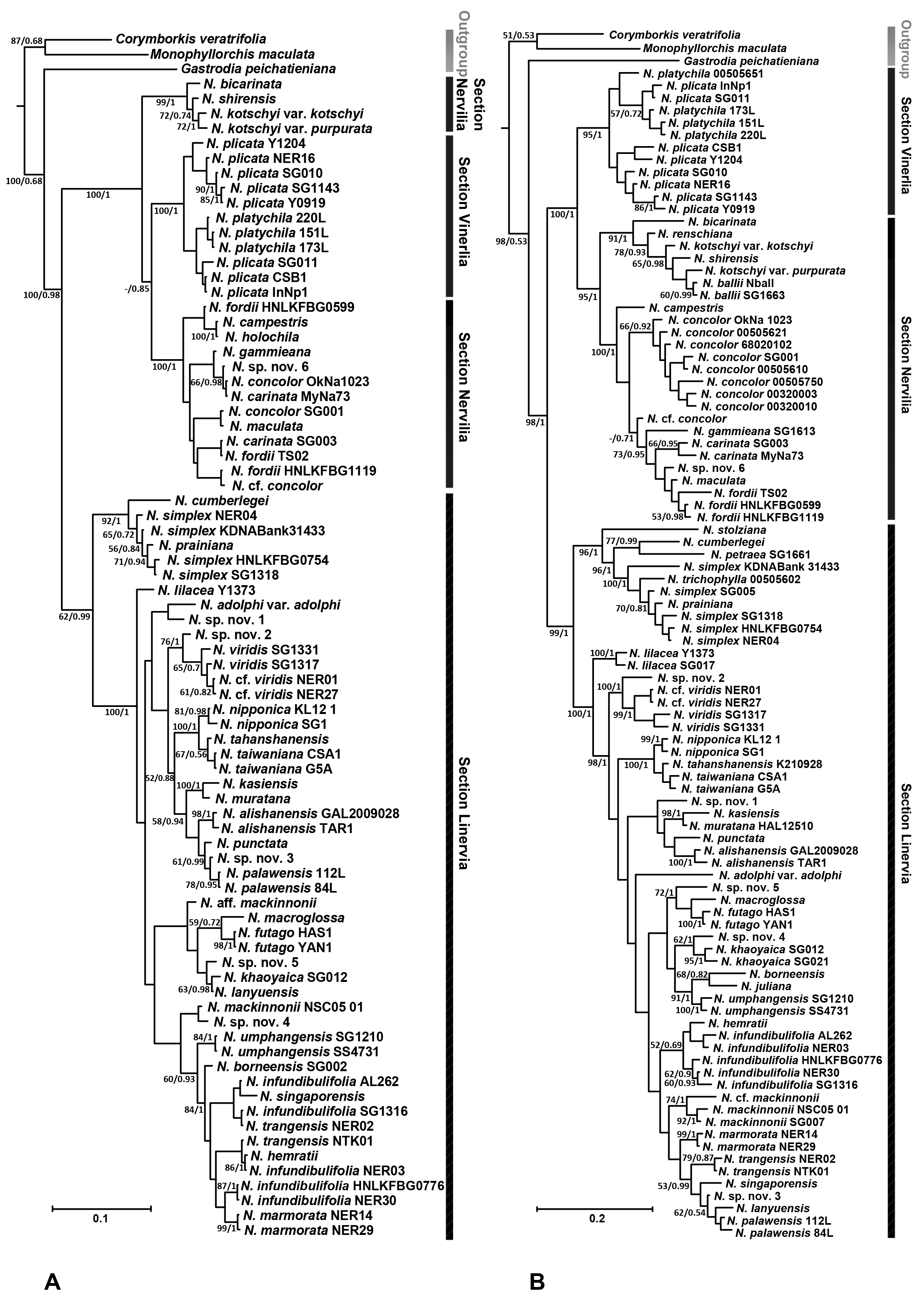

Supplement: Supplementary file 3 [file Image1.jpg]
